# Factors related to blood pressure assessment during pregnancy in Ethiopia: Multilevel analysis using the 2019 mini demographic and health survey data

**DOI:** 10.1371/journal.pone.0309247

**Published:** 2024-08-26

**Authors:** Tigabu Kidie Tesfie, Bantie Getnet Yirsaw, Muluken Chanie Agimas, Mehari Woldemariam Merid, Nebiyu Mekonnen Derseh, Werkneh Melkie Tilahun

**Affiliations:** 1 Department of Epidemiology and Biostatistics, Institute of Public Health, College of Medicine and Health Sciences, University of Gondar, Gondar, Ethiopia; 2 Department of Public Health, College of Medicine and Health Sciences, Debre Markos University, Debre Markos, Ethiopia; Arba Minch University, ETHIOPIA

## Abstract

**Introduction:**

Blood pressure assessment is an essential strategy for early detection and treatment of hypertension and hypotension. Hypertensive disorders of pregnancy (HDP) are major public health problems resulting in a significant burden of perinatal and maternal morbidity and mortality. In Ethiopia, among pregnancies complicated by HDP, 25% end up with perinatal death. Perinatal and maternal mortality related to HDP were found to be higher in Ethiopia compared to high-income and most of the low- and middle-income countries. Despite its importance, there is limited evidence on blood pressure assessment during pregnancy. Therefore, this study aimed to determine the prevalence of blood pressure assessment during pregnancy and its associated factors in Ethiopia.

**Methods:**

This study was based on the 2019 Mini Ethiopian Demographic and Health Survey data. A total weighted sample of 2923 women who had a live birth five years before the survey were included and Stata version 16 software was used for statistical analysis. To identify associated factors, a multilevel robust Poisson regression model was fitted since the prevalence of blood pressure assessment was higher than 10%. Variables with p-value < 0.2 in the bi-variable analysis were exported to the multivariable analysis. In the multivariable analysis, the adjusted prevalence ratio with its 95% confidence interval was used to declare a statistically significant association.

**Results:**

In Ethiopia, the prevalence of blood pressure assessment during pregnancy was 88.1% (95% CI: 86.9%, 89.2%). In the multivariable multilevel robust Poisson analysis, primary education and secondary education, grand-multiparity, initiation of antenatal care before three months and 3–6 months, four and above antenatal care visits, being counselled by a health professional, being from richer and richest households, residing in Afar and Amhara regions were significantly associated with BP assessment during pregnancy in Ethiopia.

**Conclusion and recommendations:**

To reduce the high burden of mortality related to hypertensive disorders of pregnancy in the country, blood pressure assessment should be improved. Therefore, policymakers should design interventions that empower women in terms of education and economy, promoting early initiation of antenatal care visits and prenatal counselling could improve blood pressure assessment.

## Introduction

Hypertensive disorders of pregnancy (HDP) are global public health problems that cause higher rates of maternal death, preterm birth, stillbirth, and neonatal death [1–5]. These disorders are the most common medical conditions during pregnancy and may complicate up to 10–15% of pregnancies worldwide [6]. According to a systematic review and meta-analysis report, the prevalence of hypertensive disorder of pregnancy in Ethiopia was 6.1% with a confidence limit of 4.8–7.3% [7]. HDP contributes to 14% and 16% of maternal deaths globally and in Sub-Saharan Africa, respectively [6, 8].

Maternal and perinatal mortality related to HDP occur disproportionately higher in low- and middle-income countries because of poor quality of maternal and child health care and lower levels of health service utilization [9, 10]. In Ethiopia, 19% of maternal mortality is attributable to HDP [11, 12]. Perinatal and maternal mortality related to HDP were found to be higher than reports from high-income and most of the low- and middle-income countries. For instance, according to a systematic review and meta-analysis report, in Ethiopia, one perinatal death occurs among four pregnancies complicated by HDP. In addition, the rate of having a low birth weight newborn is 37% [12]. Despite the availability of programs in the country including maternal care services provided during pregnancy through antenatal care [13], there is still a high prevalence of HDP, which calls for special emphasis by stakeholders and healthcare professionals.

Early identification and timely intervention can reduce maternal and perinatal deaths from HDP [6, 14]. Treatments for HDP are available in many settings. However, appropriate interventions are based on the correct recognition of hypertension [15]. After hypertension diagnosis, evidence-based intervention strategies targeting the mother and its fetus can be initiated to monitor and control the progression of the disease to its severe forms including preeclampsia, eclampsia, and HELLP syndrome (hemolysis, elevated liver enzymes, and low platelet count). So, routine BP assessment during antenatal care facilitates early diagnosis of HDP and subsequent administration of antihypertensive therapy and monitoring to prevent associated fetal and maternal complications [16]. It is therefore crucial that accurate blood pressure (BP) measurement should be done to control hypertension and its consequences. Moreover, hypotension secondary to shock and hemorrhage which is a common event during pregnancy is identified only through BP assessment [17–19]. From previous study findings, hypotensive pregnant women were found at higher risk of severe vomiting and nausea, abortion, hemorrhage, and anemia [20].

BP is a vital sign that guides short and long-term clinical decision-making. It is measured in millimeters of mercury (mmHg), the pressure exerted within the arterial walls. Conventionally it is separated into systolic and diastolic components. Systolic BP is the maximum pressure on the arterial wall during contraction of the ventricles whereas diastolic BP is the minimum pressure recorded on the arterial wall during diastole (ventricular relaxation and filling) [21, 22].

Appropriate assessment of BP during pregnancy is the cornerstone of obstetric care. It is simple, life-saving, and cost-effective. BP assessment helps to identify and treat HDP. In addition, BP assessment can identify hypotension secondary to septic shock and hemorrhage [15, 23, 24], which are among the four main causes of pregnancy-related mortality together with HDP and abortion [25]. Assessment of BP is a routine medical practice in the outpatient department and helps to follow the hemodynamic conditions of inpatients. The primary purposes of BP assessment are to screen for hypotension and hypertension and to monitor a patient’s response to antihypertensive drugs [26].

BP measurement status was found associated with age, income, family history of hypertension, and unhealthy lifestyles like high salt intake and tobacco use [19]. Strong evidence is available on the benefits of assessment for BP and effective treatment of hypertension. For instance, a 10 mmHg reduction in the systolic BP reduced the risk of stroke by 27%, coronary heart disease by 17%, heart failure by 28%, and mortality from all causes by 13% [27, 28]. BP control is only possible when an assessment of BP is done.

In the national antenatal care (ANC) guidelines of Ethiopia, BP assessment is a component of ANC packages and should be done for every pregnant woman during her visits [13]. Despite the well-understood benefit of BP assessment during pregnancy [28], there is a lack of evidence on the prevalence of BP measurement and its associated factors in Ethiopia as well as the rest of the world [29]. Determining the prevalence of BP assessment during pregnancy and the identification of possible predictors could help to improve the service by targeting contributing factors. Therefore, this study aims to determine the national prevalence of BP assessment during pregnancy and identify associated factors.

## Methods

### Study design, period, and setting

This study was conducted based on the 2019 Mini Ethiopian Demographic and Health Survey (MEDHS) data, which is a nationally representative cross-sectional study conducted from March 21 to June 28, 2019, across Ethiopia [30].

### Source and study population

The source population was all women who had ANC follow-up for their most recent live birth in the five years before the survey. The study population was all women in the selected enumeration areas who had ANC follow-up for their most recent live birth in the five years prior to the survey.

### Sampling procedure, sample size, and data source

In the 2019 MEDHS, a two-stage stratified cluster sampling technique was used. In the first stage, 305 enumeration areas (93 urban and 212 rural) were chosen based on the 2019 Ethiopian Population and Housing Census frame. In the second stage, a fixed number of 30 households in each cluster were chosen with an equal probability of systematic selection. A total of 2,935 women who had ANC for their most recent live birth in the five years prior to the survey were asked about their BP measurement history. In this study, a total of 2923 survey-weighted samples were analyzed [30]. The DHS program was our data source. The data was acquired using https://www.dhsprogram.com/data/available-datasets.cfm after submitting a summary of the rationale of the study and analysis methods.

### Variables of the study and measurement

#### Dependent variable

The outcome variable of this study was the self-reported history of BP assessment during pregnancy among women who had ANC for their most recent live birth in the five years prior to the survey (“measured” or “not-measured”). Women who had at least one BP measurement during ANC visits for their most recent live birth in the five years prior to the survey were considered "measured,” otherwise “not-measured” [30].

#### Independent variables

Possible factors were identified from the literature. According to a study from Tanzania, factors associated with BP check-ups during pregnancy include age, educational status, parity, timing of the first ANC visit, wealth index, and region [31]. A study from Ethiopia indicates that the number of ANC visits and place of residence were found to be associated with the utilisation of ANC service components [32]. In addition, according to a study from India, the sex of the household head was found associated with maternal health service utilization [33]. Furthermore, counselling status during pregnancy, marital status, religion, and place of ANC visit were additional independent variables investigated by the study.

### Data management and analysis

After accessing the data from the MEASURE DHS website, Stata version 16 was used for data extraction, recoding, and descriptive and inferential analysis. Descriptive statistics were employed and presented as text, frequency, percentage, figures, and tables. To restore the representativeness of the study we have applied sample weighting and during analysis weighted sample was used.

The MEDHS data has a hierarchical nature since women are nested within a cluster, and we expect that women within the same cluster are more similar to each other than women found in different clusters. The intra-class correlation coefficient (ICC) was estimated to measure the variability explained by cluster variation. The intra-class correlation coefficient (ICC) is the degree of within-cluster dependence or heterogeneity across clusters [34, 35]. During analysis, there was significant variability in BP assessment among pregnant women, explained by cluster variation (ICC > 10%). This violates the observation independence and equality of variance assumptions of the classical logistic regression model. This implies that we should account for cluster variability by using a mixed-effects model [36, 37].

Since the dependent variable had a binary category, a multilevel logistic regression model was expected to be applied to estimate the association between independent variables and BP measurement status. But the odds ratio (OR) is a good approximation of the prevalence ratio (PR) when the outcome is uncommon or rare (< 10%). When the prevalence is greater than 10%, as in our case, the PR can be misestimated by the OR. Hence, we employed a multilevel robust Poisson regression model to get reliable estimates [38, 39].

#### Model-building process

To identify factors associated with BP assessment, initially, chi-square assumptions were checked, and variables that fulfill the assumption were entered into a bi-variable robust Poisson regression analysis. Variables that had a p-value < 0.2 in the bi-variable analysis were exported to the multivariable analysis. Four models were constructed for the mixed-effect Poisson regression model with robust variance. The first model was an empty model without explanatory variables to determine the extent of cluster variation on BP assessment; the second model was constructed with individual-level variables; the third model was constructed with community-level variables; and the fourth model was constructed with individual and community-level variables together. Deviance and log-likelihood (LL) were used to compare and select the best-fitted model, and a model with the lowest deviance and largest LL was considered the best-fitted model. Finally, the adjusted prevalence ratio (APR) of the best-fitted model with its 95% confidence interval (CI) was reported, and variables with a P value <0.05 in the multivariable multilevel robust Poisson regression analysis were considered significant predictors of BP assessment during pregnancy.

### Ethical consideration

All methods were carried out per the relevant guidelines of the Demographic and Health Surveys (DHS) program. A letter of permission for data access was obtained from the DHS program. Publicly available data with no personal identifier was used.

## Results

### Descriptive results

A total weighted sample of 2923 women who had ANC for their most recent live birth in the five years prior to the survey was included. The mean age of respondents was 28.4 (±6.4 standard deviation) years. Regarding educational status, 43.9% of women were not educated. The majority (94.2%) of women were married. In terms of religion, 41.8% of participants were Orthodox Christians. More than half (57.8%) of the participants had ≥4 ANC visits, and more than three-fourths (78.9%) of women started their first ANC visit between 3–6 months of their pregnancy. The majority of women (94.8%) attend their ANC in governmental health facilities. Out of ten, seven women received counselling by health professionals during pregnancy. More than one-fourth of women (26.4%) live in the richest households. Regarding the sex of the household head, 86.8% of respondents came from male-headed households. Rural residents were 70.2%, and 36.8% of women were from Oromia (Table 1).

**Table 1:** Characteristics of study participants, using MEDHS 2019.

Variable	Category	Weighted frequency (n = 2923)	Percentage (%)
**Age group**	15–19	153	5.2
	20–24	606	20.7
	25–29	948	32.4
	30–34	603	20.6
	35–39	410	14.0
	40–44	157	5.4
	45–49	47	1.6
**Marital status**	Single	13	0.5
	Married	2754	94.2
	Separated/ divorced/widowed	156	5.3
**Religion**	Orthodox Christian	1223	41.8
	Catholic	6	0.2
	Protestant	793	27.1
	Muslim	876	30.0
	Traditional/other	25	0.9
**Educational status**	No education	1282	43.9
	Primary	1153	39.5
	Secondary	335	11.4
	Higher	153	5.2
**Parity**	Primiparity	689	23.6
	Multiparity	1370	46.8
	Grand-multiparity	864	29.6
**Timing of first ANC visit**	Before 3 months	348	11.9
	3–6 months	2307	78.9
	After 6 months	268	9.2
**Number of ANC visits**	<4	1235	42.2
	≥4	1688	57.8
**Place of ANC visits**	Governmental	2771	94.8
	Private/NGO	152	5.2
**Counselling status during pregnancy**	Yes	2077	71.1
	No	846	28.9
**Wealth index**	Poorest	399	13.7
	Poor	587	20.1
	Middle	589	20.1
	Rich	578	19.8
	Richest	770	26.4
**Sex of household head**	Male	2538	86.8
	Female	385	13.2
**Residence**	Urban	871	29.8
	Rural	2052	70.2
**Region**	Tigray	271	9.3
	Afar	32	1.1
	Amhara	713	24.4
	Oromia	1076	36.8
	Somali	66	2.3
	Benishangul	39	1.3
	SNNPR	559	19.1
	Gambela	17	0.6
	Harari	9	0.3
	Addis Ababa	123	4.2
	Dire Dawa	18	0.6

### Prevalence of blood pressure assessment during pregnancy

**Fig 1:**
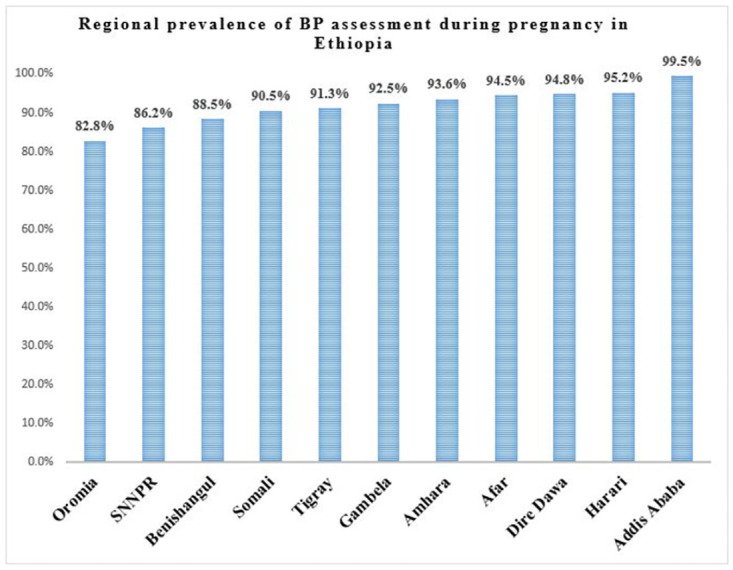
Regional prevalence of BP assessment during pregnancy in Ethiopia, using MEDHS 2019.

The overall prevalence of BP assessment during pregnancy in Ethiopia was 88.1% (95% CI: 86.9%, 89.2%). The highest and lowest BP prevalence were observed in Addis Ababa (99.5%) and Oromia (82.8%), respectively (Fig 1).

### Factors associated with BP assessment during pregnancy

#### Random effect analysis results

In the random effect robust Poisson regression analysis, the ICC value was 22.7% (95% CI: 15.5%, 32.1%), indicating that about 22.7% of the overall variability in BP assessment was explained by cluster differences while the remaining 77.3% of the variability was explained by individual differences. The likelihood ratio test (LR test mixed-effect vs. classical model: X^2^(01) = 49.74; P = 0.0000), indicates that the multilevel model better fits the data. A model comparison was made among the model without an explanatory variable (empty-model), a model including individual-level factors (Model-I), a model including community-level factors (Model-II), and a model including individual- and community-level variables (Model-III) using model comparison parameters. The model with individual and community level factors together (Model-III) had the smallest AIC, the smallest deviance, and the largest log-likelihood. Finally, factors associated with BP assessment were reported based on the best-fitted model. The ICC value was reduced to 1.1% in the final model (Table 2).

**Table 2:** Comparison between mixed-effect models using log-likelihood, deviance, and AIC.

Model	LL	Deviance	AIC
**Empty-model**	-2920.7	5841.4	5843.4
**Model-I**	-2908.1	5816.2	5856.2
**Model-II**	-2915.4	5830.8	5854.8
**Model-III**	-2906.4	5812.8	5830.7

#### Fixed-effect analysis results

In the fixed effect robust Poisson regression analysis, variables including education, parity, timing of the first ANC visit, number of ANC visits, counselling status, wealth index, and region were significantly associated with BP assessment during pregnancy.

The prevalence of BP assessment among pregnant women who had primary education increased by 5% (APR = 1.05, 95% CI: 1.02, 1.08) as compared to women who didn’t have education. The prevalence of BP assessment among pregnant women who had secondary education increased by 6% (APR = 1.06, 95% CI: 1.01, 1.09) as compared to women who didn’t have education. Regarding parity, the prevalence of BP assessment among grand-para women increased by 4% (APR = 1.04, 95% CI: 1.01, 1.08) as compared to primiparous women.

The prevalence of BP assessment was found to increase by 11% (APR = 1.11, 95% CI: 1.04, 1.19) among women who started their ANC visit before three months of the pregnancy as compared to women who started their ANC visit after six months. Women who started their ANC between three and six months had a 10% (APR = 1.10, 95% CI: 1.02, 1.17) higher prevalence of BP assessment as compared to women who started their ANC visit after six months of their pregnancy.

We found a 4% (APR = 1.04, 95% CI: 1.01, 1.06) higher prevalence of BP assessment among women who had four or more ANC visits as compared to women who had fewer than four ANC visits. Women who were counselled by health professionals had a 13% (APR = 1.13, 95% CI: 1.09, 1.16) higher prevalence of BP assessment as compared to those who weren’t counselled by health professionals.

The household wealth index significantly determines the prevalence of BP assessment. Women who had a richer household wealth index had an 8% (APR = 1.08, 95% CI: 1.03, 1.14) higher prevalence as compared to women from the poorest households. Women from the richest households had 11% (APR = 1.11, 95% CI: 1.06, 1.17) higher prevalence of BP assessment as compared to women from the poorest households.

Furthermore, the variable region was found to significantly determine the BP assessment status during pregnancy. Women from the Afar and Amhara regions had a 10% (APR = 1.10, 95% CI: 1.03, 1.17) and 5% (APR = 1.05, 95% CI: 1.001, 1.10) higher prevalence of BP measurement, respectively, as compared to women who reside in Tigray **(**Table 3).

**Table 3:** Bi-variable and multivariable mixed-effect robust poisson regression analysis, using MEDHS 2019.

Variable	Category	CPR (95% CI)	P-value	APR (95% CI)
**Age group**	15–19	1		
	20–24	0.99 (0.94, 1.05)	0.859	
	25–29	0.98 (0.93, 1.03)	0.515	
	30–34	0.99 (0.94, 1.04)	0.697	
	35–39	0.99 (0.94, 1.06)	0.996	
	40–44	1.00 (0.94, 1.08)	0.905	
	45–49	0.93 (0.81, 1.06)	0.274	
**Religion**	Orthodox	1		1
	Catholic	0.97 (0.82, 1.14)	0.715	1.01 (0.86, 1.20)
	Protestant	0.93 (0.89, 0.98)	0.003	0.98 (0.94, 1.03)
	Muslim	0.98 (0.95, 1.02)	0.313	0.99 (0.97, 1.03)
	Traditional/other	0.85 (0.67, 1.08)	0.187	0.95 (0.74, 1.21)
**Educational status**	No education	1		1
	Primary	1.06 (1.03, 1.09)	0.000	1.05 (1.02, 1.08)***
	Secondary	1.11 (1.07, 1.15)	0.000	1.06 (1.01, 1.09)***
	Higher	1.12 (1.08, 1.16)	0.000	1.03 (0.99, 1.07)
**Parity**	Primiparity	1		1
	Multiparity	0.98 (0.95, 1.01)	0.229	1.01 (0.98, 1.04)
	Grand-multiparity	0.96 (0.93, 0.99)	0.035	1.04 (1.01, 1.08)*
**Timing of first ANC visit**	Before 3 months	1.25 (1.16, 1.35)	0.000	1.11 (1.04, 1.19)**
	3–6 months	1.17 (1.09, 1.26)	0.000	1.10 (1.02, 1.17)**
	After 6 months	1		1
**Number of ANC visits**	<4	1		1
	≥4	1.10 (1.07, 1.13)	0.000	1.04 (1.01, 1.06)**
**Place of ANC visits**	Governmental	1		1
	Private/NGO	1.09 (1.06, 1.11)	0.000	1.00 (0.97, 1.03)
**Counselling status during pregnancy**	Yes	1.17 (1.13, 1.21)	0.000	1.13 (1.09, 1.16)***
	No	1		1
**Wealth index**	Poorest	1		1
	Poorer	1.00 (0.95, 1.06)	0.941	1.01 (0.96, 1.08)
	Middle	1.04 (0.99, 1.10)	0.126	1.05 (0.99, 1.11)
	Richer	1.09 (1.04, 1.14)	0.000	1.08 (1.03, 1.14)***
	Richest	1.17 (1.12, 1.21)	0.000	1.11 (1.06, 1.17)***
**Sex of household head**	Male	1		1
	Female	1.03 (1.01, 1.06)	0.018	1.01 (0.98, 1.04)
**Residence**	Urban	1		1
	Rural	0.90 (0.88, 0.92)	0.000	0.99 (0.96, 1.03)
**Region**	Tigray	1		1
	Afar	1.03 (0.97, 1.10)	0.308	1.10 (1.03, 1.17)**
	Amhara	1.03 (0.97, 1.09)	0.394	1.05 (1.001, 1.10)*
	Oromia	0.91 (0.84, 0.99)	0.023	0.94 (0.88, 1.01)
	Somali	0.99 (0.92, 1.09)	0.981	1.08 (0.99, 1.18)
	Benishangul	0.97 (0.90, 1.03)	0.293	0.99 (0.94, 1.04)
	SNNPR	0.95 (0.88, 1.02)	0.159	0.98 (0.93, 1.04)
	Gambela	1.00 (0.94, 1.07)	0.945	1.03 (0.97, 1.10)
	Harari	1.06 (0.99, 1.13)	0.090	1.03 (0.97, 1.08)
	Addis Ababa	1.10 (1.04, 1.16)	0.000	1.01 (0.97, 1.06)
	Dire Dawa	1.05 (0.98, 1.12)	0.142	1.03 (0.98, 1.09)

CPR: Crude Prevalence Ratio; APR: Adjusted Prevalence Ratio;

*p-value <0.05;

**p-value< 0.01;

***p-value<0.001

## Discussion

Increased BP among pregnant women is a worldwide public health challenge with life-threatening consequences. Early detection and treatment of hypertension are possible through appropriate BP assessment [40]. Even though evidence on the prevalence and related factors of BP measurement during pregnancy is essential for healthcare professionals, policymakers, and researchers, a study has not been conducted so far on the problem in the study area. Therefore, this study could provide a piece of essential evidence for interventions aiming to improve BP assessment and reduce the associated risk of maternal and perinatal mortality from high or low BP.

In this study, the prevalence of BP assessment among pregnant women in Ethiopia was 88.1% (95% CI: 86.9%, 89.2%) ranging from 82.8% in Oromia to 99.5% in Addis Ababa. The higher prevalence of BP assessment observed in Addis Ababa might be because it is the capital city of the country where more educated and rich people who have better awareness of and access to ANC services live [41]. Although BP assessment during pregnancy is a part of comprehensive ANC service packages, still 11.9% of pregnant women weren’t assessed for BP status during ANC follow-up in Ethiopia. The prevalence of BP assessment during pregnancy in Ethiopia is found higher than that of Tanzania which was 72.2% [31]. Study setting and period differences might be the possible reasons for the discrepancy.

Better women’s educational status was found significantly and positively associated with a higher prevalence of BP assessment. Women with primary and secondary education had a 5% and 6% increased prevalence of BP measurement, respectively, compared to women without education. This finding is supported by a study done in Tanzania [31], and a systematic review that showed a positive association between being educated and the utilisation of ANC services in Ethiopia [42]. This might be due to the positive impact of education on improving maternal health service-seeking behavior, understanding of the benefits of using ANC services, and enhancing a woman’s control over her pregnancy [43–46]. In addition, educated women tend to have a better understanding of HDP and its consequences which propels them to seek screening for hypertension during pregnancy. Previous studies observed adequate knowledge about HDP among women with better education [47, 48]. Furthermore, women who had higher education tend to have adequate knowledge about the necessary measures during pregnancy and services to take from health facilities [49].

The prevalence of BP assessment among grand-multipara women was significantly increased by 4% as compared to primiparous women. Multiparous women are more likely to have previous exposure to prenatal services including BP assessment which makes them claim for BP assessment during ANC visits. Having better experience in ANC service utilization could have a positive impact on the women’s level of knowledge and practice about ANC as observed in a study done in Eritrea [50]. On the contrary, studies conducted in China [51, 52] and Tanzania [31, 53] reported that high-parity women were less likely to utilize ANC services. The possible explanation might be that high-parity women are more likely to ignore the need for ANC services and solely depend on their own experiences from previous pregnancies [54].

The prevalence of BP assessment was found to increase among women who initiate their ANC visits early. This finding is supported by a study conducted in Tanzania [31]. Women who start their ANC early (timely) can have more numbers of ANC visits which increases the possibility of BP assessment during pregnancy and taking recommended services as indicated by a study from Malawi [55]. In addition, based on a study finding from Ethiopia, early initiation of ANC visits was found significantly and positively associated with adequate numbers of ANC visits [56]. According to the evidence at hand, women who had four and above numbers of ANC visits had an increased prevalence of BP assessment.

Counselling status by health professionals was found significantly associated with BP assessment during pregnancy. Those women who were counselled by health professionals were more likely to have BP assessments. This might be due to the positive roles of prenatal counselling. Prenatal counselling might provide a safe space for the woman to share concerns about her pregnancy with the health professional which might bolster the health professional to measure the woman’s BP, increase knowledge of pregnant women about hypertension screening, and help to make informed decisions [57]. According to a study done in Indonesia [58], counselling during ANC visit was found to improve the knowledge and attitude of pregnant women about pregnancy danger signs.

Belonging to the richer and richest household wealth index was found significantly associated with increased prevalence of BP assessment as compared to women from the poorest households. According to a study done in Ethiopia, as the household wealth index increases the frequency of ANC service utilization was found to increase [59]. This finding is also supported by a study conducted in Indonesia [44] and a systematic review and meta-analysis conducted in Sub-Saharan Africa [54] that showed the positive relationship between better wealth index and maternal health service utilisation. In addition, our study finding was consistent with a study done in Tanzania in which richer women were more likely to check their BP during ANC as compared to poorer women [31]. This finding might be due to the reason that women who belong to households with higher wealth index usually had better access to mass media [60, 61], higher educational status [62], and enhanced decision-making power [63] which improves a woman’s BP assessment status during the prenatal follow-up period.

The prevalence of BP assessment was significantly increased in the Amhara and Afar regions as compared to the Tigray region. Regional variations of BP assessment among pregnant women were also observed in a study conducted in Tanzania [31]. A study conducted in Ethiopia also found higher regional disparities in ANC utilization across the country [41]. This finding might be due to geographical differences and variations in the quality, access, and coverage of maternal and child healthcare services in the country [64]. This finding calls for the equitable distribution of resources necessary for BP assessment across regions.

### Strengths and limitations of the study

The strengths of the study are as follows: first, this is the first study to analyze the prevalence of BP assessment and identify its associated factors during pregnancy in Ethiopia. So, this study findings will help to understand the depth of the problem and its determinants, and capture researchers’ interest for further exploration. Secondly, the representativeness of the study was restored using weighted samples during analysis. However, the limitations are: to begin with, institutional and health professional characteristics that might influence BP assessment were not incorporated since we have used secondary data. It was a bottleneck for us to identify facility level and health professional-related factors that possibly determine BP assessment during pregnancy. In addition, the study might be prone to recall and social desirability biases for the reason that it is solely based on the verbal response of the women. Recall bias might under or overestimate the prevalence of BP assessment whereas social desirability bias might overestimate the prevalence of BP assessment. So, interpretations of the study findings should consider these limitations.

## Conclusion and recommendations

Even though most pregnant women in Ethiopia had a BP assessment at least once during ANC follow-up, 11.9% of women were never assessed for BP. To reduce the high burden of HDP-related mortality in the country, BP assessment prevalence should be improved. Therefore, policymakers should design interventions that empower women in terms of education and the economy. Hence, intersectoral collaboration might be important in designing policies and plans to improve BP assessment practice. The equitable distribution of important resources for BP assessment could be applied to reduce regional disparities in service utilization. Promoting early ANC visit initiation, optimum number of ANC visits, and prenatal counselling could improve BP assessment practice. Future studies incorporating institutional and health professional characteristics might be important to identify additional factors related to BP assessment.

## Abbreviations

ANC: Antenatal Care
APR: Adjusted Prevalence Ratio
BP: Blood Pressure
CI: Confidence Interval
CPR: Crude Prevalence Ratio
DHS: Demographic and Health Survey
HDP: Hypertensive Disorders of Pregnancy
ICC: Intra-class Correlation Coefficient
MEDHS: Mini Ethiopian Demographic and Health Survey
NGO: Non-Governmental Organizations
SNNPR: South Nations Nationalities and Peoples Region
VIF: Variance Inflation Factor
